# Locust Pathogen *Aspergillus oryzae* XJ1 Is Different from *Aspergillus oryzae* and *Aspergillus flavus* Based on Genomics Comparisons

**DOI:** 10.3390/microorganisms12122501

**Published:** 2024-12-04

**Authors:** Yinwei You, Xiao Xu, Hui Liu, Long Zhang

**Affiliations:** Shandong Key Laboratory for Green Prevention and Control of Agricultural Pests, Institute of Plant Protection, Shandong Academy of Agricultural Sciences, Jinan 250100, China; yyw30000@163.com (Y.Y.); xuxiao_cau@yeah.net (X.X.); liuhui6892@gmail.com (H.L.)

**Keywords:** *Aspergillus*, genome, comparison, biological control, locust pathogen, core/pan-genome analysis

## Abstract

Fungi play an increasingly important role in the biological control of insect pests. *Aspergillus oryzae* XJ1 is highly virulent to locust adults and nymphs, which are a destructive economic pest worldwide. Because of its host association with locusts, which is unique in *Aspergillus*, in this study, we examined the genetic relationships of *A. oryzae* XJ1 within *Aspergillus*. We sequenced the genome of *A. oryzae* XJ1 and compared it with the genomes of other *Aspergillus* species. The complete genome of *A. oryzae* XJ1 is 37.9 Mb, comprising 11,720 putative genes, assembled into eight chromosomes. The genome size is similar to that of other *A. oryzae* strains. Phylogenomic analysis indicated that *A. oryzae* XJ1 was most closely related to *A. flavus* NRRL3357, not *A. oryzae* RIB40. Core/pan-genome analysis of *A. oryzae* XJ1 and other *Aspergillus* species revealed that *A. oryzae* XJ1 had 704 strain-specific genes, whereas *A. flavus* NRRL3357, *A. oryzae* KDG 21, and *A. parasiticus* NRRL 2999 had 646, 955, and 779 unique genes, respectively. The *A. oryzae* XJ1 genome showed structural differences compared with the genomes of *A. oryzae* RIB40 and *A. flavus* NRRL3357 in genomic synteny analysis. These results indicate that *A. oryzae* XJ1 is genetically distinct at the genome level from other *Aspergillus* species, including *A. oryzae* and *A. flavus*, and may be as a distinct species. This will provide new insight into the classification of *Aspergillus* based on genomics.

## 1. Introduction

Approximately 1000 known species of entomopathogenic fungi infect most insect species [1]. To date, ~13 species or subspecies of insect-pathogenic fungi have been formulated and registered as pesticides [2]. These products mainly comprise *Metarhizium anisopliae* and *Beauveria bassiana*, the most popularly used entomopathogenic fungi. Reports of the development of *Aspergillus* spp. as biopesticides are extremely rare.

Recently, *A. oryzae* XJ1 was identified based on morphological characteristics and molecular analysis of the sequence of 18S rDNA and reported to be highly virulent to locust adults and nymphs, both in laboratory bioassays and large-scale field experiments [3,4]. Thus, this fungal strain shows promise for the biological control of locusts. The finding that *A. oryzae* XJ1 is an entomopathogenic fungus is in strong contrast to other strains of *A. oryzae*, which is a non-entomopathogenic species and has been used for centuries in the production of traditional fermented food products, such as soy sauce, soybean paste, and rice wine, in China, Japan, and other Asian countries [5]. Several reports indicate that *A. flavus*, *A. fumigutus*, and *A. niger* are capable of infecting certain locusts, including *Schistocerka gregaria*, *Oxya velox*, *Poekilocerus pictus*, *Hieroglyphus nigrorepletus*, *Hieroglyphus perpolita*, and *Hieroglyphus oryzivorous* [6,7,8].

To study the functions of genes, characters of genome and molecular phylogeny between the species or strains in *Aspergillus*, so far, at least 104 genomes of different *A. oryzae* strains have been sequenced [9,10,11,12]. Intraspecific genome comparisons between *A. oryzae* RIB326 and *A. oryzae* RIB40, and between *A. oryzae* and *A. flavus* and other species have been performed previously [13,14]. And these results revealed genetic, metabolic and phenotypic variation within *A. oryzae* and between *A. oryzae* and *A. flavus*. However, recent pan-genome analysis between *A. oryzae* and *A. flavus* highlighted their phylogenetic, genomic, and metabolic homogeneity, potentially indicating that they may belong to the same species [15]. The results challenge the conventional view which considers *A. flavus* and *A. oryzae* distinct species. There are some different conclusions on the species of *A. flavus* and *A. oryzae* from the genome-level analysis. This also raises our question of whether *A. oryzae* XJ1 is similar to *A. flavus* or not.

In this study, to examine genome differences between *A. oryzae* XJ1 and other *Aspergillus* species, we conducted whole-genome sequencing and analyzed the genome profile of *A. oryzae* XJ1 in comparison with other *Aspergillus* species. The results revealed that *A. oryzae* XJ1 is genetically distinct from other strains of *A. oryzae* and *A. flavus* at the genome level, and may be a distinct species.

## 2. Materials and Methods

### 2.1. Strain Culture

*Aspergillus oryzae* XJ1 was inoculated in potato dextrose agar liquid medium and cultured on a shaker at 28.0 ± 1.0 °C for 2 days; then, the mycelium was collected for extraction of genomic DNA.

### 2.2. Genome Sequencing of A. oryzae XJ1

#### 2.2.1. Extraction of Genomic DNA of *A. oryzae* XJ1

Genomic DNA of *A. oryzae* XJ1 was extracted using the sodium dodesyl sulfate method [16]. The purified DNA quality was checked by agarose gel electrophoresis and quantified with a Qubit^®^ 2.0 Fluorometer (Thermo Fisher Scientific, Waltham, MA, USA).

#### 2.2.2. Library Construction of *A. oryzae* XJ1

(1) PacBio Sequel platform (Pacific Biosciences, Menlo Park, CA, USA)

Libraries for single-molecule real-time (SMRT) sequencing were constructed with an insert size of 20 kb using the SMRTbell™ Template Prep Kit 1.0 (Pacific Biosciences, Menlo Park, CA, USA). Briefly, the procedure comprised fragmentation and concentration of the DNA, damaged DNA was repaired and fragment ends were polished, blunt ligation of the fragment ends was performed, the SMRTbell templates were purified with 0.45× AMPure^®^ PB Beads (Beckman Coulter, Inc., Pasadena, CA, USA), size selection was conducted using the BluePippin™ System (Sage Science, Beverly, MA, USA), and DNA damage was repaired after size selection. Finally, the library quality was assessed with a Qubit 2.0 Fluorometer (Thermo Fisher Scientific, Waltham, MA, USA) and the insert fragment size was detected with an Agilent 2100 Bioanalyzer (Agilent Technologies, Palo Alto, CA, USA).

(2) Illumina NovaSeq platform (Illumina, Inc., San Diego, CA, USA)

A total amount of 1 μg DNA per sample was used as input material for the DNA sample preparations. Sequencing libraries were generated using the NEBNext^®^ Ultra™ DNA Library Prep Kit for Illumina (New England Biolabs, Ipswich, MA, USA) following the manufacturer’s recommendations and index codes were added to attribute sequences to each sample. Briefly, the DNA sample was fragmented by sonication into 350 bp fragments, and the fragments ends were polished, A-tailed, and ligated with the full-length adaptor for Illumina sequencing with further PCR amplification. Finally, the PCR products were purified using the AMPure XP system (Beckman Coulter, Inc., Pasadena, CA, USA), and libraries were analyzed for size distribution with an Agilent 2100 Bioanalyzer (Agilent Technologies, Palo Alto, CA, USA) and quantified using real-time PCR.

#### 2.2.3. Sequencing

The whole genome of *A. oryzae* XJ1 was sequenced using the PacBio Sequel and Illumina NovaSeq PE150 platforms by the Beijing Novogene Bioinformatics Technology Co., Ltd (Beijing, China).

#### 2.2.4. Genome Assembly

##### Preliminary Assembly with SMRT Link 5.0.1 software suite (Pacific Biosciences, Menlo Park, CA, USA)

(1) To ensure the reliability of the subsequent analysis, the low-quality reads (less than 500 bp) were discarded to obtain the clean data.

(2) Using the automatic error correction function accessible on the SMRT Portal (Pacific Biosciences, Menlo Park, CA, USA), the long reads (more than 6000 bp) were selected as the seed sequence, and the other shorter reads were aligned to the seed sequence with BLASR v.3.1.0 to enable the accuracy of the seed sequence to be improved further. This process yielded the preliminary assembly.

##### Correction of the Preliminary Assembly

Using the Structural Variant Calling module of the SMRT Link 5.0.1 software suite, the Arrow algorithm was used to correct and count the variant sites in the preliminary assembly.

#### 2.2.5. Genome Component Prediction

Genome component prediction involved the prediction of the coding genes, repetitive sequences, and non-coding RNA. The steps were performed as follows.

(1) By default, we used the Augustus 2.7 program to retrieve the relevant coding genes for fungi. If homologous reference gene sequences and transcript sequencing data were available, a complete annotation pipeline, PASA, as implemented at the Broad Institute, involved the following steps: (A) ab initio gene finding using a selection of the following software tools: GeneMarkHMM (http://opal.biology.gatech.edu/GeneMark/, accessed on 25 November 2021), FGENESH (http://www.softberry.com/berry.phtml?topic=case_study_animal&no_menu=on, accessed on 25 November 2021), Augustus 2.7, SNAP version 2006-07-28, and GlimmerHMM 3.0.2; (B) protein homology detection and intron resolution using the GeneWise software wise2-4-1 and the uniref90 non-redundant protein database; (C) alignment of known expressed sequence tags, full-length cDNAs, and Trinity RNA-Seq assemblies to the genome; (D) PASA alignment assemblies were based on overlapping transcript alignments from step (C); (E) use of EVidenceModeler (EVM) (https://github.com/EVidenceModeler/EVidenceModeler, accessed on 25 November 2021) to compute weighted consensus gene structure annotations based on the preceding steps (A–D); and (F) use of PASA to update the EVM consensus predictions, adding untranslated region annotations and models for alternatively spliced isoforms (leveraging D and E).

(2) The interspersed repetitive sequences were predicted using RepeatMasker (http://www.repeatmasker.org/, accessed on 25 November 2021). The tandem repeats were analyzed with the Tandem Repeats Finder program 4.07b.

(3) Transfer RNA (tRNA) genes were predicted with the tRNAscan-SE version1.3.1. Ribosomal RNA (rRNA) genes were analyzed using rRNAmmer V1.2. Small RNA (sRNA), small nuclear RNA (snRNA), and microRNAs (miRNA) were predicted by performing a BLAST search against the Rfam database.

#### 2.2.6. Prediction of Gene Functions

We used seven databases to predict gene functions, namely, GO (Gene Ontology), KEGG (Kyoto Encyclopedia of Genes and Genomes), KOG (Clusters of Orthologous Groups), NR (Non-Redundant Protein Database), TCDB (Transporter Classification Database), P450, and Swiss-Prot. A whole-genome BLAST search (E-value < 1 × 10^−5^, minimal alignment length percentage > 40%) was performed against each of the databases. Secretory proteins were predicted with the Signal P database. We analyzed the secondary metabolism gene clusters using antiSMASH V4.0.2. For pathogenic fungi, we added pathogenicity and drug resistance information using the PHI (Pathogen Host Interactions) and DFVF (Database of Fungal Virulence Factors) databases. Carbohydrate-active enzymes were predicted with the CAZy (Carbohydrate-Active enZYmes) Database.

### 2.3. Comparative Genomic Analysis

#### 2.3.1. Gene Family Analysis

Gene families were constructed with the genes extracted from the genome assemblies for *Aspergillus oryzae* XJ1, *Aspergillus oryzae* RIB40 (GCA_000184455.3), *Aspergillus fumigatus* Af293 (GCA_000002655.1), *Aspergillus niger* CBS 513.88 (GCA_000002855.2), *Aspergillus terreus* NIH2624 (GCA_000149615.1), *Aspergillus luchuensis* IFO 4308 (GCA_016861625.1), *Aspergillus flavus* NRRL3357 (GCA_014117465.1), *Aspergillus nidulans* FGSC A4 (GCA_000149205.2), *Aspergillus parasiticus* ASM95608v1 (GCA_000956085.1), and *Saccharomyces cerevisiae* S288C (GCA_000146045.2). BLAST was used for pairwise alignment of all genes and redundancy was eliminated using SOLAR 0.9.6. Gene-family clustering was based on the alignment results obtained with the hcluster_sg software 0.5.0.

#### 2.3.2. Phylogenomic Tree Reconstruction Based on Genome Alignment

Alignment of the *A. oryzae* XJ1 genome and the aforementioned nine reference genomes was performed using the MUMmer 3.23 and LASTZ tools 1.03.54. A phylogenomic tree was constructed using the maximum likelihood algorithm with TreeBeST 1.9.2. The reliability of the tree topology was assessed by performing a bootstrap analysis was 1000 replicates.

#### 2.3.3. Core/Pan-Genome Analysis

Core genes and strain-specific genes between the *A. oryzae* XJ1 genome and nine reference genomes [*Aspergillus oryzae* RIB40 (GCA_000184455.3), *Aspergillus oryzae* KDG21 (GCA_018140735.1), *Aspergillus fumigatus* Af293 (GCA_000002655.1), *Aspergillus niger* CBS 513.88 (GCA_000002855.2), *Aspergillus terreus* NIH2624 (GCA_000149615.1), *Aspergillus luchuensis* IFO 4308 (GCA_016861625.1), *Aspergillus flavus* NRRL3357 (GCA_014117465.1), *Aspergillus nidulans* FGSC A4 (GCA_000149205.2), and *Aspergillus parasiticus* NRRL 2999 (GCA_012897115.1)] were analyzed using the CD-HIT software 4.6 for rapid clustering of similar proteins with a threshold of 50% pairwise identity and 0.7 length difference cutoff. A Venn diagram was generated to visualize the proportions of core and unique genes among the strains.

#### 2.3.4. Genomic Synteny Analysis

Genomic synteny between *A. oryzae* XJ1 and *A. oryzae* RIB40, and between *A. oryzae* XJ1 and *A. flavus* NRRL3357 was analyzed based on the aforementioned genome alignment.

## 3. Results

### 3.1. General Information on the A. oryzae XJ1 Genome

**Figure 1:**
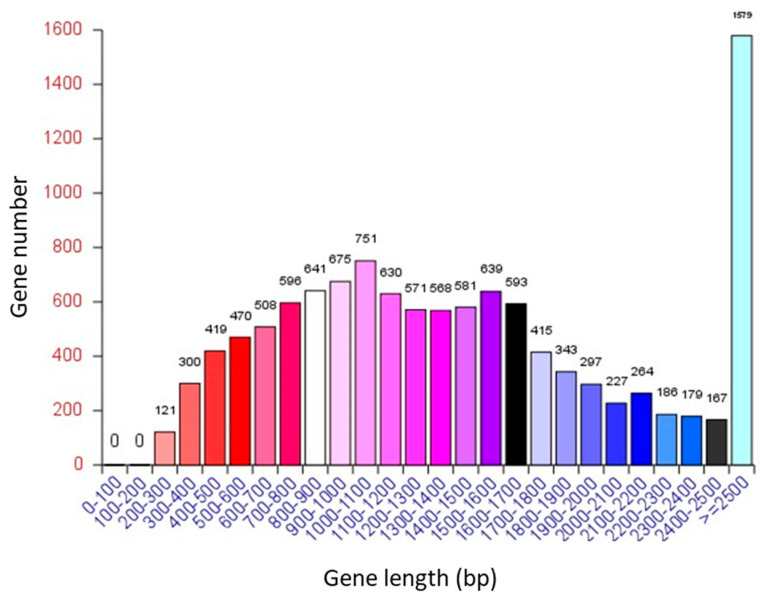
Distribution of gene length of *A. oryzae* XJ1.

The complete genome assembly for *A. oryzae* XJ1 comprised 37.874054 Mbp and encoded 11,720 predicted genes with an average length of 1557 bp (Table 1). The total length of genes was 18.250593 Mbp, comprising 48.19% of the total genome length, whereas the gene internal length was 19.623461 Mbp. The GC content of the genes was 52.09%, compared with 42.88% for the gene internal GC content (Table 1). The number of genes of length ≥ 2500 bp was 1579, much higher than that of other lengths, which comprised 13.47% of the total number of genes (Figure 1).

**Figure 2:**
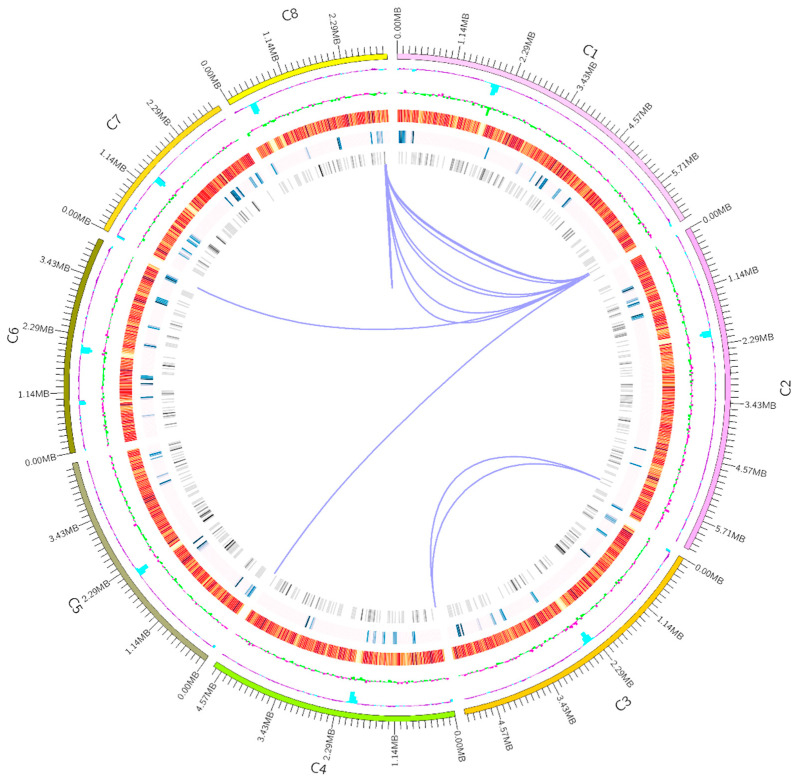
Circular representation of the complete genome of *Aspergillus oryzae* XJ1. DNA sequencing revealed that the genome of *A. oryzae* XJ1 comprised eight chromosomes. The outermost circle shows the chromosome size (kb) for each chromosome (C1–C8). The second circle from the outside shows the GC content for each chromosome; inward blue boxes indicate a lower GC content than the average, purple boxes indicate a higher GC content than the average; the higher the peak, the larger the difference from the mean. The third circle from the outside represents GC skew values (G−C/G+C); inward green boxes indicate a mean G content lower than C, pink boxes indicate a mean G content higher than C. The fourth circle from the outside indicates the distribution of coding genes. The fifth circle shows the distribution of pathogenicity genes. The sixth circle indicates the genes associated with metabolism. The innermost circle shows chromosome duplication.

A total of 10 scaffolds were identified and assigned to chromosomes 1 to 8 (Figure 2). The length of each chromosome was predicted as follows: C1, 6,525,071 bp; C2, 6,309,536 bp; C3, 5,185,625 bp; C4, 4,749,098 bp; C5, 4,562,565 bp; C6, 4,149,163 bp; C7, 3,211,970 bp; C8, 3,161,472 bp; C9, 14,541 bp; and C10, 5013 bp. Because C9 and C10 were so short, they are not included in the genome structure in Figure 2.

### 3.2. Analysis of Predicted Gene Functions in A. oryzae XJ1

**Figure 3:**
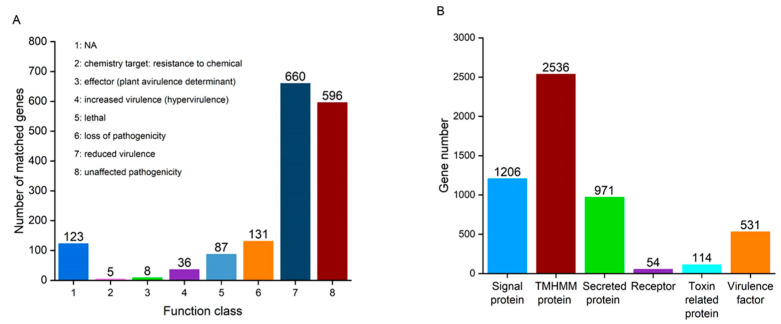
Genes involved in host–pathogen interactions in signaling pathways (**A**) and virulence factors (**B**) in the *Aspergillus oryzae* XJ1 genome.

The predicted genes in the *A. oryzae* XJ1 genome were annotated with pathway information from the KEGG database. Of the annotated genes, 596 genes were predicted to be associated with unaffected pathogenicity, 660 genes with reduced virulence, 131 genes with loss of pathogenicity, 87 genes with lethality, 36 genes with increased virulence, 8 genes were effectors, 5 genes were associated with resistance to chemicals, and 123 genes had no annotated function using the PHI database (Figure 3A). In total, 1646 predicted genes were related to pathogenicity.

Signaling pathways and toxins may be important in interactions between the host and the pathogen. In the *A. oryzae* XJ1 genome, 1206 genes encoded signaling proteins, 54 genes encoded receptors, 971 genes encoded secreted proteins, and 2536 genes encoded TMHMM proteins (Figure 3B). Toxin and virulence factors, as lethality-related factors, may play important roles in the death of the host. Regarding virulence-related factors in the *A. oryzae* XJ1 genome, 114 genes encoded toxin-related proteins and 531 genes encoded virulence factors.

### 3.3. Evolutionary Analysis of the A. oryzae XJ1 Genome

**Figure 4:**
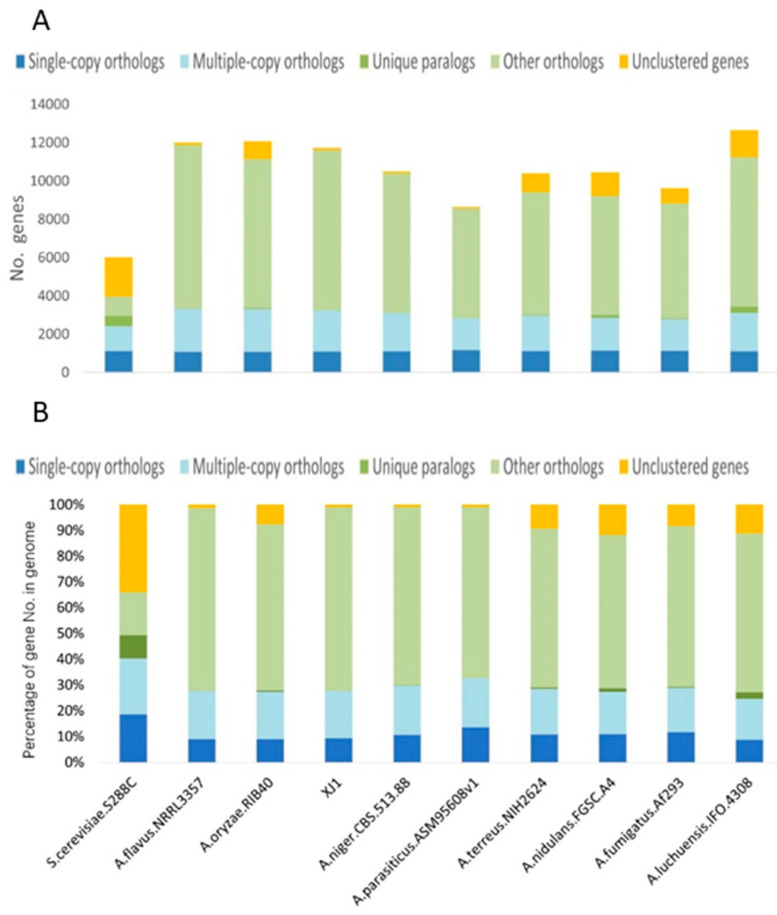
Number (**A**) and proportion (**B**) of orthologous genes in nine *Aspergillus* strains compared with *Saccharomyces cerevisiae*.

The total number of genes in the *A. oryzae* XJ1 genome was lower than that of *A. flavus* NRRL3357 and *A. oryzae* RIB40 (Figure 4A). The numbers of single-copy orthologs, multiple-copy orthologs, and non-clustered genes of *A. oryzae* XJ1 were similar to those of *A. flavus* NRRL3357. The numbers of single-copy orthologous genes were extremely similar among the 10 fungal strains analyzed, whereas for other gene clusters, such as multiple-copy orthologs, unique paralogs, other orthologs, and non-clustered genes, the number of genes varied among these species.

Comparison of the proportions of the different groups of orthologous genes revealed that the pattern in *A. oryzae* XJ1 was very similar to that of *A. flavus* NRRL3357, *A. niger* CBS 513.88, and *A. parasiticus* ASM95608v1, and less so to that of *A. oryzae* RIB40, *A. fumigatus* Af293, and *A. terreus* NIH2624 (Figure 4B).

**Figure 5:**
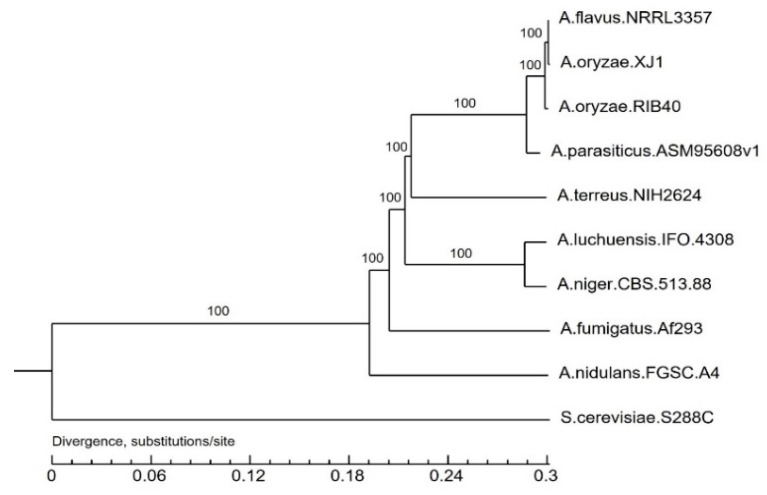
Phylogenomic relationships of *Aspergillus oryzae* XJ1 within the *Aspergillus* genus.

To analyze phylogenomic relationships, a phylogenetic tree was constructed from an alignment of the genomes for the 10 strains. The number of substitutions/site for *A. flavus* NRRL3357 (0.000746995), *A. oryzae* XJ1 (0.00116078), *A. oryzae* RIB40 (0.00223712), *A. parasiticus* ASM95608v1 (0.00904747), *A. terreus* NIH2624 (0.0871863), *A. luchuensis* IFO 4308 (0.013228), *A. niger* CBS 513.88 (0.0130487), *A. fumigatus* Af293 (0.0974948), *A. nidulans* FGSC.A4 (0.109048), and *S. cerevisiae* S288C (0.301509) indicated that *A. oryzae* XJ1 was most closely related to *A. flavus* NRRL3357 and slightly more distantly related to *A. oryzae* RIB40 (Figure 5).

**Figure 6:**
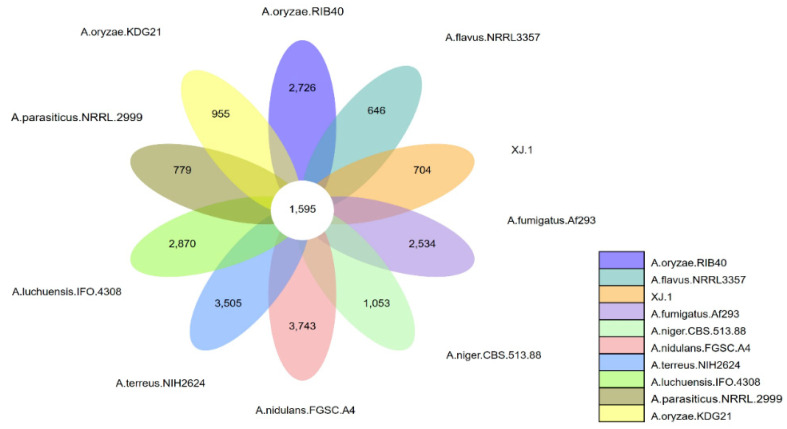
Core/pan-genome analysis of genes in the genomes of eight species of *Aspergillus*.

The core/pan-genome analysis revealed that the *A. oryzae* XJ1 genome included 704 unique genes, whereas *A. flavus* NRRL3357, *A. oryzae* KDG21, and *A. parasiticus* NRRL 2999 had 646, 955, and 779 strain-specific genes, respectively. The genomes of the remaining six fungal species had more than 1000 strain-specific genes (Figure 6). These ten *Aspergillus* strains had 1595 common genes (Figure 6).

**Figure 7:**
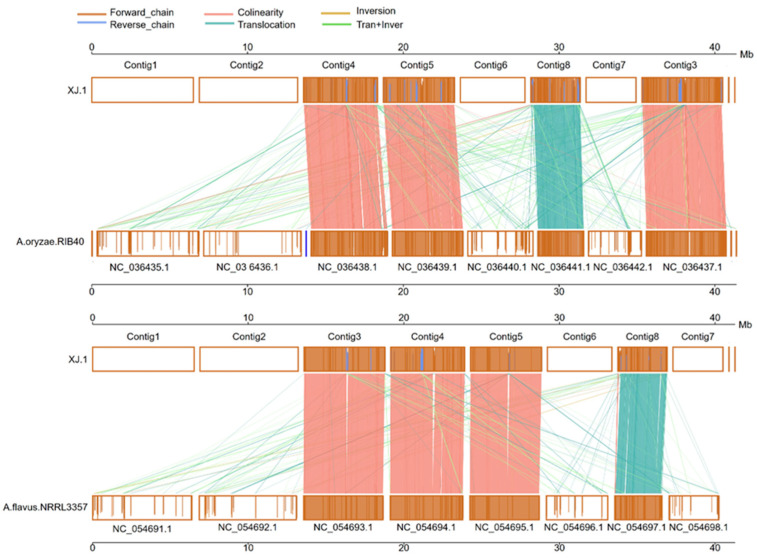
Analysis of core genes in the genomes of *Aspergillus oryzae* XJ1, *A. flavus* NRRL3357, and *A. oryzae* RIB40. The shading color in boxes indicates the degree of similarity between the genomes; the deeper the color, the higher the similarity.

The *A. oryzae* XJ1 genome showed structural differences compared with the genomes of *A. oryzae* RIB40 and *A. flavus* NRRL3357 (Figure 7). The *A. oryzae* XJ1 genome contained three contigs with an almost colinear forward chain (brown color and pink color) and one contig with translocation genes (darker green color) compared with both *A. flavus* NRRL3357 and *A. oryzae* RIB40. A greater number of reverse chains were present in *A. oryzae* XJ1 compared with *A. oryzae* RIB40 than in comparison with *A. flavus* NRRL3357 (blue color). A greater number of inversion genes (saffron color) and translocation+inversion genes (green color) were detected in *A. oryzae* XJ1 compared with *A. oryzae* RIB40 than in comparison with *A. flavus* NRRL3357.

## 4. Discussion

A previous study indicated that *A. oryzae* XJ1 was most similar to strains of *A. oryzae* in morphology [3]. Its colony color was yellowish brown and olive-green, light-yellow and olive-green on Czapek’s agar medium after 30, 10 days at 25 °C, respectively. Its conidia surface was smooth [3]. These characteristics indicated that the strain *Aspergillus sp*. XJ1 was *A. oryzae*, not *A. flavus*. In the present study, a comparison of the *A. oryzae* XJ1 genome to that of nine *Aspergillus* strains indicated that *A. oryzae* XJ1 was genetically distinct at the genome level from other *Aspergillus* species, including two *A. oryzae* strains and one *A. flavus* strain. Although the genome size of *A. oryzae* XJ1 was similar to that of *A. oryzae* RIB40 and *A. flavus* NRRL3357, at approximately 37 Mb, the numbers of putative genes differed, i.e., 11,720, 12,074, and 13,485, respectively [17,18]. The proportions of the different groups of orthologous genes in *A. oryzae* XJ1 were very similar to the patterns observed for *A. flavus* NRRL3357 and *A. oryzae* RIB40. However, the core/pan-genome analysis, phylogenomic reconstruction, and genomic synteny analysis indicated that *A. oryzae* XJ1 was genetically distinct from *A. flavus* and *A. oryzae* when compared with other genomes of *Aspergillus* genus.

Previously, *Aspergillus flavus* and *A. oryzae* were considered two different species. But these two species are, in fact, very similar. Numerous studies have sought to distinguish them, including on the basis of morphological characteristics [19], amplified fragment length polymorphisms [20], restriction fragment length polymorphisms [21], sequences of the nuclear ribosomal internal transcribed spacer (ITS) region [22], assessment of aflatoxin gene clusters [23], analysis of the *cyp51A* gene [24], and matrix-assisted laser desorption/ionization time-of-flight mass spectrometry analysis [25], in addition to a study integrating selective culture methods, microscopic observations, secondary metabolite profiles, and ITS sequences [26]. However, these approaches have yet to provide an unambiguous means of differentiation between *A. flavus* and *A. oryzae*. Recent research suggests that *A. flavus* and *A. oryzae* should be one species based on analyses of phylogenetic, genomic, and metabolic homogeneity [15]. *Aspergillus oryzae* has been suggested to be a domesticated ecotype of *A. flavus* that is unable to produce the most potent natural carcinogen, aflatoxin, and any other carcinogenic metabolites [27,28]. *Aspergillus oryzae* is classified as Generally Recognized as Safe (GRAS) by the Food and Drug Administration in the USA, which is also supported by the World Health Organization [29,30,31]. It has occasionally been reported that *A. oryzae* shows pathogenicity to animals. However, surprisingly, our previous bioassay experiments showed that *A. oryzae* XJ1 is highly virulent to nymphs and adults of *Locusta migratoria* and other species of locusts [3,4]. The corrected reduction rate could peak at 94.9 ± 5.1% on the 17th day after treatment in a large-scale field trial in 2022 [4]. We found two genes, one gene is 642 bp, another gene is 669 bp, encoding insecticidal crystal toxin, P42, from the data of *A. oryzae* XJ1 genome and transcriptome data, which may be lethal to locusts. For entomopathogenic fungi, the host specificity of virulence (representing a unique environmental niche) should be a crucial consideration in the recognition of species in the genus. *Aspergillus oryzae* XJ1 has the specific ability to kill locusts. Therefore, we propose that recognition of *A. oryzae* XJ1 as a new species distinct from *A. flavus* and *A. oryzae* is warranted, with specific virulence to acridids similar to *Metarhizium acridum* [32,33]. To characterize this strain, more studies should be conducted in molecule biology, pathogenicity, ecology and developmental biology in the future.

That *A. oryzae* XJ1 could effectively kill adult locusts is very important for new biopesticide development. Previously, the main used locust pathogens were *Metarhizium anisopliae*, *Beauveria bassiana* and *Nosema locustae*, which were less effective to adult locusts [34,35]. Adult is an important stage harmful to agriculture, as adults feed on more food, have greater capacity of migration or dispersal to cross countries, and cause locust plagues cross-regionally. When locust adult swarms appeared, only chemical pesticides could be chosen to apply. This control action caused chemical pesticide pollution to both agricultural products and environments. Therefore, the further research and development of *A. oryzae* XJ1 will be an alternative to control locust adult swarms. It has been demonstrated that *A. oryzae* XJ1 can infect and kill at least 16 species of adult locusts, including *L. migratoria*, *Calliptamus italicus*, *Calliptamus abbreviates*, *Oedaleus asiticus*, *Ceracris kiangsu*, *Epacromius coerulipes*, *E. tergestinus*, *Oxya chinensis*, *Atractomorpha sinensis*, *Acrida cinerea*, *Aiolopus tamulus*, *Calliptamus barbarous*, *Oedipoda miniata*, *Sphingonotus coerulipes*, *Oedaleus decorus*, and *Notostaurus albicornis* in lab, making it a bio-control agent with high potential for developing biological control for the whole locust life cycle in the future.

To know its pathogenic mechanisms, five space mutants of *A. oryzae* XJ1, TQ201, TQ238, TQ302, TQ549, and TQ555 had previously been screened [34]. The virulence of TQ549 was significantly higher than that of *A. oryzae* XJ1, and the virulence of TQ302 was significantly lower than that of *A. oryzae* XJ1 [36]. And the comparisons between the genomes of these five space mutants and the transcriptomes of different treated *A. oryzae* XJ1 will be helpful to screen pathogenic genes and to reveal molecular pathogenic mechanisms. In fact, some candidate pathogenic genes have been found, including two insecticidal crystal toxins, P42, as mentioned above. But it needs to do more work to characterize their functions. These will not only enrich the understanding of pathogenic mechanisms of insecticidal fungi, but also lay a solid foundation for better developing and applying adult locust bio-control agent.

## 5. Conclusions

(1) The complete genome assembly for *A. oryzae* XJ1 comprised 37.874054 Mbp and encoded 11,720 predicted genes. The GC content of the genes was 52.09%, compared with 42.88% for the gene internal GC content. The genome comprised eight chromosomes, just like other *A. oryzae*.

(2) In total, there were 1646 predicted genes related to pathogenicity in *A. oryzae* XJ1 genome when using the PHI database to screen. In total, 114 predicted genes encoded toxin-related proteins and 531 predicted genes encoded virulence factors.

(3) Phylogenetic tree and comparison of the proportions of the different groups of orthologous genes both revealed that *A. oryzae* XJ1 was more closely related to *A. flavus* NRRL3357 than *A. oryzae* RIB40. The core/pan-genome analysis revealed that the *A. oryzae* XJ1 genome included 704 unique genes. The *A. oryzae* XJ1 genome showed structural differences compared with the genomes of *A. oryzae* RIB40 and *A. flavus* NRRL3357.

(4) Combined with these results, we conclude that *A. oryzae* XJ1 is different from *Aspergillus oryzae* and *Aspergillus flavus*, and may be a distinct species.

## Figures and Tables

**Table 1:** General information of *A. oryzae* XJ1 genome.

Genome Information Summary	
Genome size (bp)	37,874,054
GC content of genome (%)	47.31
Gene number	11,720
Gene total length (bp)	18,250,593
GC content of gene (%)	52.09
Gene length/genome (%)	48.19
Gene average length (bp)	1557
Gene internal length (bp)	19,623,461
Gene internal GC content (%)	42.88
Gene internal length/genome (%)	51.81

## Data Availability

The original contributions presented in this study are included in the article. Further inquiries can be directed to the corresponding author.
